# Dietary Flavonoids as Therapeutics for Preterm Birth: Luteolin and Kaempferol Suppress Inflammation in Human Gestational Tissues *In Vitro*

**DOI:** 10.1155/2013/485201

**Published:** 2013-06-05

**Authors:** Courtney Wall, Ratana Lim, Marin Poljak, Martha Lappas

**Affiliations:** Department of Obstetrics and Gynaecology, University of Melbourne, Mercy Hospital for Women, Heidelberg, Melbourne, VIC 3084, Australia

## Abstract

Infection/inflammation is commonly associated with preterm birth (PTB), initiating uterine contractions and rupture of fetal membranes. Proinflammatory cytokines induce matrix metalloproteinases (MMPs) that degrade the extracellular matrix (ECM) and prostaglandins which initiate uterine contractions. Nuclear factor-**κ**B (NF-**κ**B) and activator-protein- (AP-)1 have key roles in the formation of these prolabour mediators. In nongestational tissues, dietary flavonoids such as luteolin and kaempferol inhibit NF-**κ**B, AP-1, and their downstream targets. The aim of this study was to determine if luteolin and kaempferol reduce infection-induced prolabour mediators in human gestational tissues. Fetal membranes were incubated with LPS, and primary amnion cells and myometrial cells were incubated with IL-1*β* in the absence or presence of luteolin or kaempferol. Luteolin and kaempferol significantly reduced LPS-induced secretion of proinflammatory cytokines (IL-6 and IL-8) and prostaglandins (PGE_2_ and PGF_2**α**_) in fetal membranes, IL-1*β*-induced COX-2 gene expression and prostaglandin production in myometrium, and IL-1*β*-induced MMP-9 activity in amnion and myometrial cells. Luteolin and kaempferol decreased IL-1*β*-induced NF-**κ**B p65 DNA binding activity and nuclear c-Jun expression. In conclusion, luteolin and kaempferol inhibit prolabour mediators in human gestational tissues. Given the central role of inflammation in provoking preterm labour, phytophenols may be a therapeutic approach to reduce the incidence of PTB.

## 1. Introduction

Preterm birth (PTB) is the single most important complication contributing to poor pregnancy and neonatal outcome, globally, being defined as childbirth occurring at less than 37-week gestation. According to the World Health Organisation (WHO), more than 1 in 10 babies is born preterm every year [1], and this incidence has been steadily rising since the 1980s despite obstetric intervention [2]. With long term health consequences, the care of the prematurely born infant is extremely expensive, and the emotional stress on the family is sizeable. The estimated cost associated with PTB in the United States alone, in terms of medical and educational expenditure and lost productivity in 2005, was more than US$26.2 billion [3].

Spontaneous PTB (sPTB) accounts for approximately 70% of all PTB with 60% due to idiopathic preterm labour and the remaining 40% due to preterm pre-labour rupture of the fetal membranes (PPROM) [4]. Infection is the biggest aetiological factor for the onset of sPTB [5]. Infection activates the maternal immune system, which causes production of the proinflammatory cytokines IL-1β and TNF-*α*. They bind to their respective receptors located on placenta, fetal membranes, and myometrium to induce the activity of the proinflammatory and prolabour transcription factors activator-protein- (AP-)1 and nuclear factor kappa B (NF-*κ*B) [6–9]. Once activated, AP-1 and NF-*κ*B upregulate transcription of proteases, prostaglandins, and cytokines which lead to delivery of the baby by causing cervical ripening, rupture of fetal membranes, and uterine contractions, three critical stages in the initiation of labour.

Epidemiological studies have revealed that a diet rich in plant-derived foods has a protective effect on human health [10, 11]. Luteolin is a citrus flavonoid found in high amounts in parsley, thyme, peppermint, basil, herb, celery, and artichoke. Kaempferol is a flavonoid found in many edible plants including tea, broccoli, cabbage, kale, beans, endive, leek, tomato, strawberries, and grapes. Previous studies performed in nongestational tissues have demonstrated that luteolin and kaempferol are two phytophenols that have potent anti-inflammatory properties, exerting these actions via inhibition of NF-*κ*B [12–15] and AP-1 [14, 16, 17]. The effect of luteolin and kaempferol as modulators of the inflammatory response associated with labour is, however, not yet known. Thus, the aim of this study is to determine whether luteolin and kaempferol will reduce infection-induced prolabour mediators in human gestational tissues. Experiments will be performed in the presence of LPS or IL-1β as these are likely to be factors stimulating proinflammatory cytokines, prostaglandins, and proteases in the context of infection-induced PTB. We analysed the effect of luteolin and kaempferol on (i) COX-2 and subsequent PGE_2_ and PGF_2*α*_ production, (ii) proinflammatory cytokine (IL-6) and chemokine (IL-8) mRNA expression and release, and (iii) matrix-metalloproteinase- (MMP-)9 mRNA expression and release. The effect of luteolin and kaempferol on NF-*κ*B (NF-*κ*B p65 DNA binding activity and I*κ*B-*α* expression) and AP-1 (nuclear c-Jun expression) was also examined.

## 2. Methods and Materials

### 2.1. Tissue Collection

Human placentae and attached fetal membranes were obtained (with Institutional Research and Ethics Committee approval) from nonobese women who delivered single, healthy infants at term gestational age (37–40-week gestation) undergoing elective Caesarean section, whether due to a medical or obstetrical reason or on patients request. Human myometrium was obtained from the upper margin of the incision made in the lower uterine segment at the time of term Caesarean section. Amnion and underlying choriodecidua were obtained 2 cm from the periplacental edge. None of the patients were in labour or had received uterotonics or tocolytics.

### 2.2. Fetal Membrane Explants

Fetal membranes (combined amnion and choriodecidua) were obtained within ten minutes of delivery, and dissected fragments were placed in ice-cold PBS. Tissue fragments were placed in Roswell Park Memorial Institute (RPMI) 1640 media at 37°C in a humidified atmosphere of 8% O_2_ and 5% CO_2_ for 1 h. Explants were blotted dry on sterile filter paper and transferred to 24-well tissue culture plates (200 mg wet weight/well). The explants were incubated, in duplicate, in 2 mL RPMI 1640 containing penicillin G (100 U/mL) and streptomycin (100 *μ*g/mL). Explants were incubated, for 24 h, in the presence of 10 *μ*g/mL LPS (to facilitate the production of proinflammatory mediators) in the absence (DMSO control) or presence of 20 *μ*M luteolin or 100 *μ*M kaempferol (Sigma, St. Louis, MO, USA) (*n* = 8 patients). A dose response was used to determine the concentrations of luteolin and kaempferol used for this study (data not shown), with the initial concentrations for the dose response determined from past studies [18–23]. After 24 h incubation, medium was collected and stored at −80°C until assayed for cytokine and prostaglandin concentrations as detailed below. Tissue was collected and stored at −80°C until assayed for gene expression by qRT-PCR. Experiments were performed in fetal membranes from eight patients.

### 2.3. Myometrial Cell Culture

Primary myometrial smooth muscle cells were used to investigate the effects of luteolin and kaempferol on the COX-prostaglandin pathway and MMP-9. Myometrial tissue was washed in PBS and finely dissected. Myometrium was minced and digested for 45 min in Dulbecco's Modified Eagle's Medium: nutrient mixture F12 (DMEM/F12) with 3 mg/mL type 1 collagenase (Worthington Biochemical, Freehold, NJ, USA) and 80 *μ*g/mL DNase I (Roche Diagnostics Australia). Cells were centrifuged at 400× g for 10 min and grown in a 75 cm^2^ flask in DMEM/F12 with 10% heat inactivated fetal calf serum (FCS), 2 mM L-glutamine, 100 U/mL penicillin, and 100 *μ*g/mL streptomycin (37°C and 5% CO_2_ and 21% O_2_). Myometrial cells from passages 1–4 were trypsinised in TrypLE Express (Life technologies, Grand Island, NY, USA) and cultured in 12-well plates in DMEM/F12 with 10% heat inactivated FCS, 2 mM L-glutamine, 100 U/mL penicillin, and 100 *μ*g/mL streptomycin until they reached the required confluence (~90%). Cells were then incubated in 500 pg/mL IL-1β (to facilitate the production of proinflammatory mediators) and either in the absence (DMSO control) or presence of 20 *μ*M luteolin or 100 *μ*M kaempferol (*n* = 6 patients) for 24 h. For I*κ*B-*α* studies, cells were pretreated with luteolin and kaempferol overnight, followed by a 30 min incubation with 500 pg/mL IL-1β. For c-Jun studies, cells were pretreated for 6 h with luteolin and kaempferol followed by an overnight incubation with 500 pg/mL IL-1β. The media were collected and stored at −80°C, until assayed for cytokine, prostaglandin, and MMP-9 concentrations as detailed below. Cell pellets were collected and stored at −80°C, before being analysed for I*κ*B-*α* and c-Jun expression by Western blotting, gene expression by qRT-PCR, or NF-*κ*B p65 DNA binding activity by transcription factor assay as detailed below. Experiments were performed in myometrium from six patients.

### 2.4. Primary Amnion Cell Culture

Primary amnion epithelial cultures were used to investigate the effects of luteolin and kaempferol on MMP-9 expression and enzyme activity. Cells were prepared as previously described [24]. Primary amnion cells (passage 1) at ~90% confluence were incubated in the absence or presence of 1 ng/mL IL-1β in the absence or presence of 20 *μ*M luteolin or 100 *μ*M kaempferol (*n* = 6 patients). After 24 h incubation, medium was collected, and assessment of enzymes of ECM weakening and rupture (MMP-9) was performed as detailed below. Cells were also collected and MMP-9 gene expression analysed by qRT-PCR as detailed below. Experiments were performed in amnion from six patients.

### 2.5. Cytokine and Prostaglandin Assays

Conditioned medium from cell and tissue culture experiments was assessed for IL-6 and IL-8 concentrations using commercial ELISA according to the manufacturer's instructions (Invitrogen, Carlsbad, CA, USA). The release of PGE_2_ and PGF_2*α*_ into the incubation medium was assayed using commercially available competitive enzyme immunoassay kits according to the manufacturer's specifications (Kookaburra Kits from Sapphire Bioscience, Redfern, NSW, Australia). All data were corrected for total protein and expressed as either ng or pg per mg protein. The protein content of tissue homogenates was determined using BCA protein assay (Pierce, Rockford, USA), using BSA as a reference standard, as previously described [25–27].

### 2.6. Gelatin Zymography

Assessment of enzymes of ECM weakening and rupture (MMP-9) was performed by gelatin zymography as previously described [27, 28]. Proteolytic activity was remodeled as clear zones of lysis on a blue background of undigested gelatin.

### 2.7. RNA Extraction and qRT-PCR

Total RNA from cells and tissues was extracted using TRI Reagent according to manufacturer's instructions (Sigma-Aldrich, St. Louis, MO, USA). RNA concentrations were quantified using a spectrophotometer (NanoDrop, Thermo Scientific). RNA quality and integrity were determined via the A_260_/A_280_ ratio. Two hundred ng (fetal membranes) or 300 ng (amnion and myometrial cells) of RNA was converted to cDNA using the SuperScript VILO cDNA Synthesis Kit (Invitrogen, Carlsbad, CA, USA) according to the manufacturer's instructions. The cDNA was diluted fiftyfold, and 4 *μ*L of this was used to perform RT-PCR using Sensimix Plus SYBR green (Quantace, Alexandria, NSW) and 100 nM of primers: GAPDH (QT01192646), IL-6 (QT00083720), IL-8 (QT00000322), TNF-*α* (QT01079561), COX-2 (QT00040586), and MMP-9 (QT00040040) (Qiagen, Germantown, MD, USA). The specificity of the product was assessed from melting curve analysis. RNA without reverse transcriptase during cDNA synthesis as well as PCR reactions using water instead of template showed no amplification. Average gene C_T_ values were remodeled to the average GAPDH mRNA C_T_ values of the same cDNA sample. Fold differences were determined using the comparative C_T_ method and expressed relative to basal [29].

### 2.8. Western Blotting

Western blotting was performed as we have previously described [25, 30]. For I*κ*B-*α* protein expression, cell lysates were prepared as detailed in [25, 27]. To assess c-Jun expression, nuclear protein was extracted as we have previously described [31]. Rabbit polyclonal anti-I*κ*B-*α* and rabbit polyclonal anti-c-Jun (Santa Cruz Biotechnology, Santa Cruz, CA, USA) were used at 1 *μ*g/mL. Forty micrograms (I*κ*B-*α*) or 5 *μ*g (c-Jun) of protein was separated on polyacrylamide gels (Bio-Rad Laboratories, Hercules, CA, USA) and transferred to PVDF. Protein expression was identified by comparison with the mobility of protein standard. Membranes were viewed and analysed using the ChemiDoc system (Bio-Rad Laboratories, Hercules, CA, USA). For the I*κ*B-*α* blot, the membranes were stripped and reprobed with β-actin (A5316; Sigma, St. Louis, MO, USA), used at 1.5 *μ*g/mL to ensure even protein loading. For the c-Jun blot, the membrane was stained with Ponceau S to ensure even loading [32].

### 2.9. NF-*κ*B p65 Transcription Factor Assay

Myometrial cells were pretreated with 20 *μ*M luteolin and 100 *μ*M kaempferol for 6 h, followed by 24 h treatment with 500 pg/mL IL-1β (*n* = 4 patients). Nuclear protein was extracted [31] and NF-*κ*B p65 DNA binding in the nuclear protein assessed using a commercially available NF-*κ*B p65 transcription factor ELISA according to manufacturer's instructions (Cayman Chemical Company, Ann Arbor, MI, USA). A Bio-Rad xMark Microplate Spectrophotometer was used to read the sample absorbance at 450 nm, with data expressed as absorbance at 450 nm.

### 2.10. Statistical Analysis

All statistical analyses were undertaken using GraphPad Prism (GraphPad Software, La Jolla, CA, USA). Data were first logarithmically transformed, before analysed by one-way ANOVA using Tukey multiple range tests. Statistical difference was indicated by a *P* value of less than 0.05. Data are expressed as mean ± standard error of the mean (SEM).

## 3. Results

### 3.1. Effect of Luteolin and Kaempferol on Proinflammatory Cytokines

**Figure 1:**
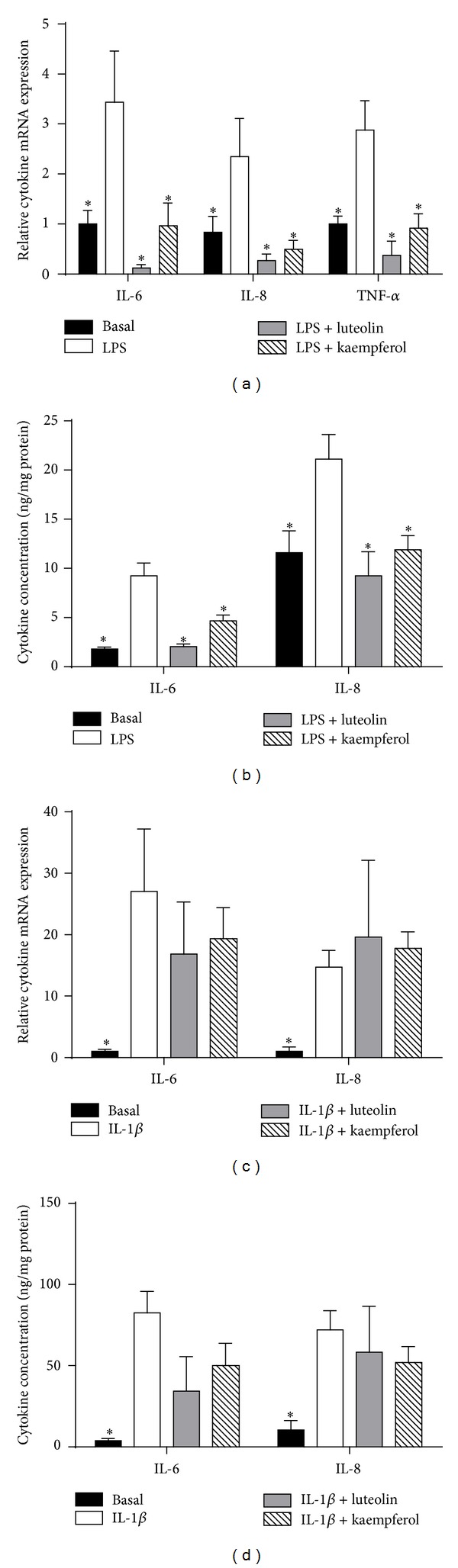
Effect of luteolin and kaempferol on proinflammatory cytokines. ((a), (b)) Fetal membranes were incubated with 10 *μ*g/mL LPS in the absence or presence of 20 *μ*M luteolin or 100 *μ*M kaempferol for 24 h (*n* = 8 patients). (a) TNF-*α*, IL-6, and IL-8 mRNA expression were quantified by qRT-PCR. (b) IL-6 and IL-8 concentrations in the conditioned media were assayed using ELISA. Each bar represents the mean ± SEM. *P < 0.05 versus basal (one-way ANOVA). ((c), (d)) Primary myometrial cells were incubated with 500 pg/mL IL-1β in the absence or presence of 20 *μ*M luteolin or 100 *μ*M kaempferol for 24 h (*n* = 6 patients). (c) IL-6 and IL-8 mRNA expressions were quantified by qRT-PCR. (d) IL-6 and IL-8 concentrations in the conditioned media were assayed using ELISA. Each bar represents the mean ± SEM. *P < 0.05 versus basal (one-way ANOVA).

In fetal membranes, qRT-PCR was performed to determine if luteolin and kaempferol reduced steady state levels of proinflammatory cytokines. As shown in Figure 1(a), LPS induced a significant increase in proinflammatory cytokine expression. Coincubation with luteolin or kaempferol significantly abrogated LPS-induced gene expression of TNF-*α*, IL-6, and IL-8. LPS induced a significantly higher concentration of both IL-6 and IL-8, when compared with basal concentration (Figure 1(a)). ELISA was then used to determine cytokine release. Coincubation with luteolin or kaempferol caused a significant reduction in LPS-induced IL-6 and IL-8 concentrations (Figure 1(b)). TNF-*α* concentration in the incubation media was below the limit of detection of the ELISA (data not shown).

Primary myometrial cells incubated with IL-1β were associated with significantly increased mRNA expression (Figure 1(c)) and release of IL-6 and IL-8 (Figure 1(d)). However, coincubation with luteolin or kaempferol had no effect on IL-1β-induced cytokine release or gene expression.

### 3.2. Effect of Luteolin and Kaempferol on the COX-Prostaglandin Pathway

**Figure 2:**
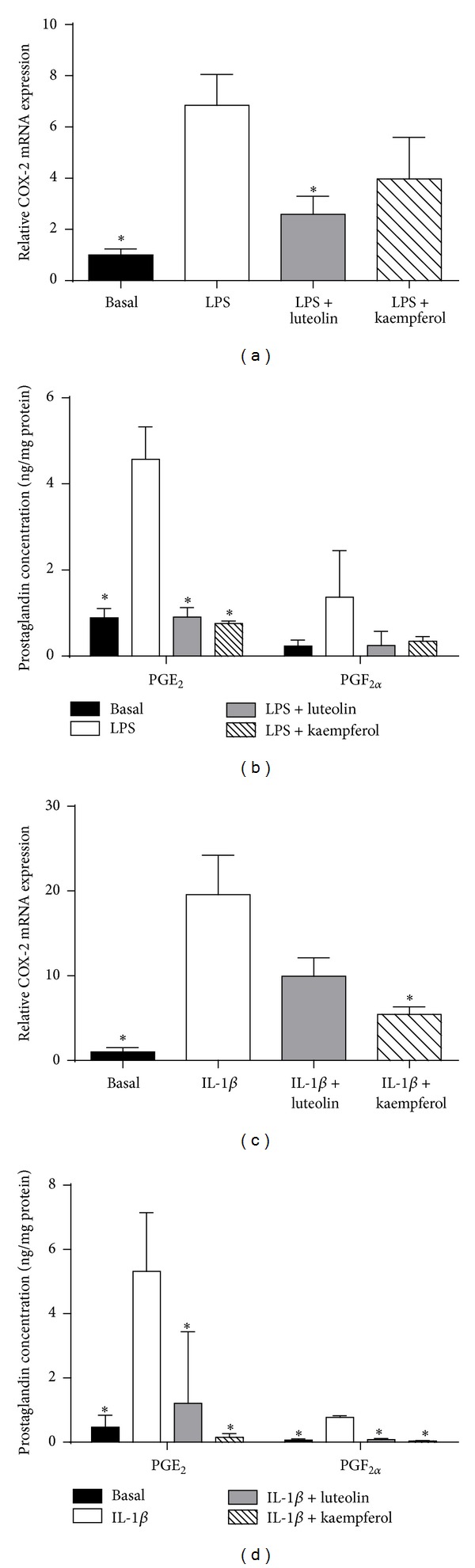
Effect of luteolin and kaempferol on the COX-prostaglandin pathway. ((a), (b)) Fetal membranes were incubated with 10 *μ*g/mL LPS in the absence or presence of 20 *μ*M luteolin or 100 *μ*M kaempferol for 24 h (*n* = 6 patients). (a) COX-2 mRNA expression was quantified by qRT-PCR. (b) PGE_2_ and PGF_2*α*_ concentrations in the conditioned media were assayed using EIA. Each bar represents the mean ± SEM. *P < 0.05 versus basal (one-way ANOVA). ((c), (d)) Primary myometrial cells were incubated with 0.5 ng/mL IL-1β in the absence or presence of 20 *μ*M luteolin or 100 *μ*M kaempferol for 24 h (*n* = 5 patients). (c) COX-2 mRNA expression was quantified by qRT-PCR. (d) PGE_2_ and PGF_2*α*_ concentrations in the conditioned media were assayed using EIA. Each bar represents the mean ± SEM. *P < 0.05 versus basal (one-way ANOVA).

Fetal membranes and myometrial cells were used to determine the effect of luteolin and kaempferol on COX-2 expression and prostaglandin levels. Experiments were performed as detailed above. When compared to basal, LPS-induced COX-2 mRNA levels (Figure 2(a)) and subsequent PGE_2_ and PGF_2*α*_ (Figure 2(b)) concentrations were significantly greater in fetal membranes. Coincubation with luteolin and kaempferol significantly decreased LPS-induced PGE_2_ concentration (Figure 2(b)). LPS-stimulated concentrations of COX-2 mRNA expression (Figure 2(a)) and PGF_2*α*_ (Figure 2(b)) were statistically decreased by luteolin but not by kaempferol.

In myometrial cells incubated with IL-1β, COX-2 mRNA levels (Figure 2(c)) and subsequent PGE_2_ and PGF_2*α*_ levels (Figure 2(d)) were significantly augmented. The addition of luteolin or kaempferol significantly attenuated IL-1β-induced PGE_2_ and PGF_2*α*_ concentrations (Figure 2(d)). However, only kaempferol reduced COX-2 mRNA expression (Figure 2(c)).

### 3.3. Effect of Luteolin and Kaempferol on MMP-9 Expression and Activity

**Figure 3:**
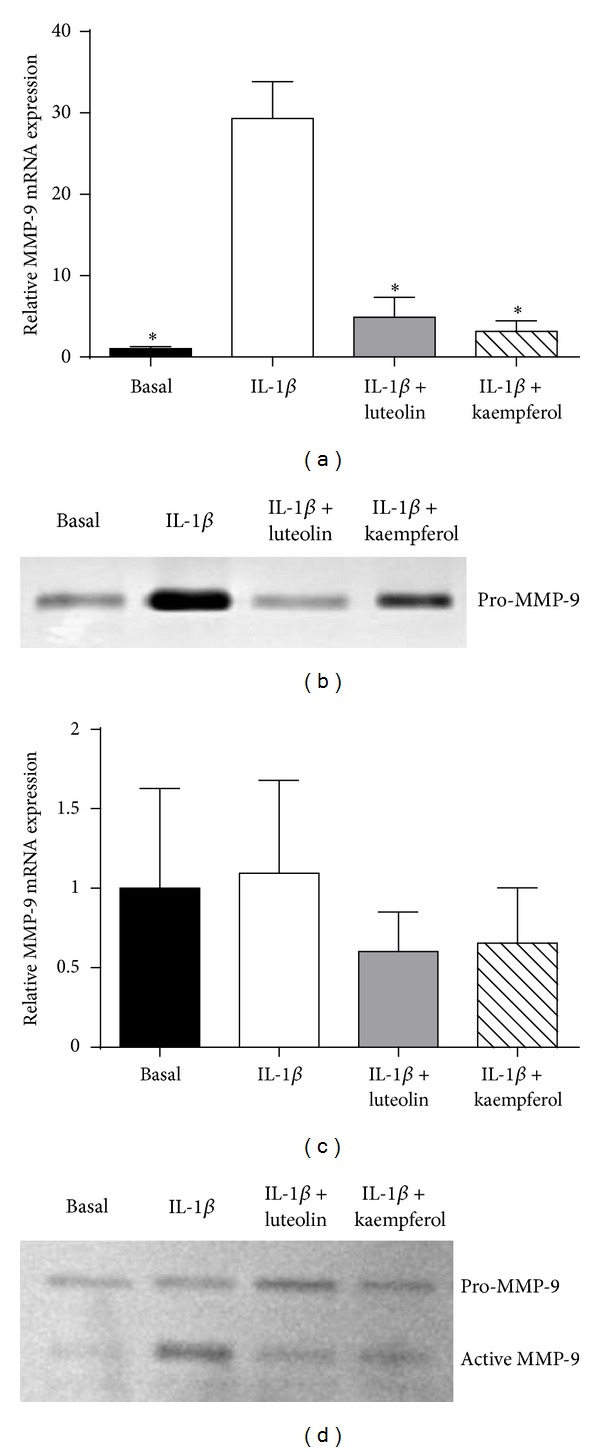
Effect of luteolin and kaempferol on MMP-9 expression and activity. ((a), (b)) Primary amnion cells were incubated with 1 ng/mL IL-1β in the absence or presence of 20 *μ*M luteolin or 100 *μ*M kaempferol for 24 h (*n* = 6 patients). ((c), (d)) Primary myometrial cells were incubated with 500 pg/mL IL-1β in the absence or presence of 20 *μ*M luteolin or 100 *μ*M kaempferol for 24 h (*n* = 4 patients). ((a), (c)) MMP-9 mRNA expression was quantified by qRT-PCR. ((b), (d)) The incubation medium was assayed for MMP-9 activity by gelatin zymography. Zymography from one patient is shown.

For amnion cells, IL-1β increased MMP-9 gene expression (Figure 3(a)) and pro-MMP-9 activity (Figure 3(b)). Coincubation with luteolin or kaempferol significantly attenuated both IL-1β-induced MMP-9 activity and expression. In myometrial cells, there was no change seen in the pro-MMP-9 bands; however there was increased active MMP-9 seen with the addition of IL-1β (Figure 3(d)). Both luteolin and kaempferol attenuated this IL-1β-induced increase in active MMP-9 activity. There was no change in MMP-9 mRNA expression with the addition of IL-1β in myometrial cells.

### 3.4. Effect of Luteolin and Kaempferol on NF-*κ*B and AP-1 Transcriptional Pathways

**Figure 4:**
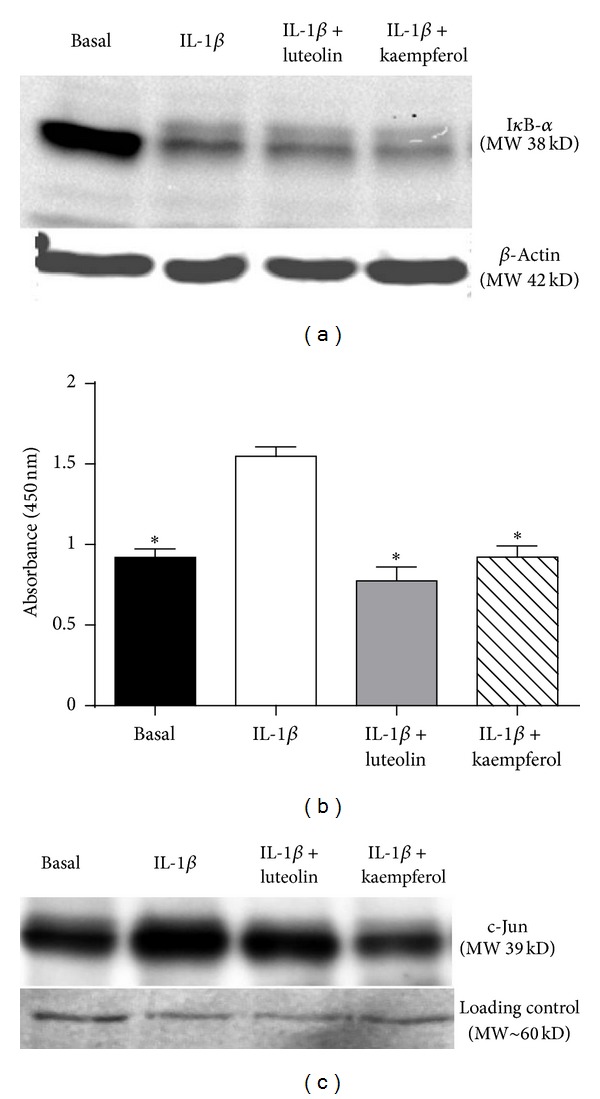
Effect of luteolin and kaempferol treatment on NF-*κ*B DNA binding activity and c-Jun expression. ((a), (c)) Primary myometrial cells were pretreated with 20 *μ*M luteolin or 100 *μ*M kaempferol for 24 h, followed by 30 min incubation with 500 pg/mL IL-1β. (a) Representative I*κ*B-*α* Western blot from one patient using β-actin as a loading control. Similar data were obtained for another two patients. (b) Primary myometrial cells were pretreated with 20 *μ*M luteolin or 100 *μ*M kaempferol for 6 h, followed by treatment with 500 pg/mL IL-1β for 24 h. NF-*κ*B p65 DNA binding activity was assayed using a NF-*κ*B p65 transcription factor assay (*n* = 4 patients). Data is displayed as absorbance at 450 nm. Each bar represents the mean ± SEM. *P < 0.05 versus IL-1β-induced NF-*κ*B DNA binding (one-way ANOVA). (c) Representative Western blot of nuclear c-Jun from one patient using Ponceau S as a loading control. Similar data were obtained for another four patients.

In unstimulated cells, the NF-*κ*B complex is made up of the p50/p65 subunits attached to I*κ*B-*α* in the cytosol. While I*κ*B-*α* is attached to the complex, NF-*κ*B is inactive. Activation by cytokines causes I*κ*B-*α* to dissociate from the NF-*κ*B complex. I*κ*B-*α* is subsequently ubiquitinated and then degraded, allowing the NF-*κ*B p50/p65 subunits to translocate to the nucleus [33]. The effect of luteolin and kaempferol treatment on IL-1β-induced I*κ*B-*α* expression was determined in myometrial cells by Western blot analysis. Myometrial cells were pretreated with 20 *μ*M luteolin and 100 *μ*M kaempferol overnight, followed by 30 min 500 pg/mL IL-1β treatment (*n* = 3 patients). As expected, IL-1β induced a decrease in I*κ*B-*α* expression (Figure 4(a)). However pre-treatment with either luteolin or kaempferol had no significant effect on attenuating this IL-1β-induced decrease in I*κ*B-*α* expression (Figure 4(a)).

Once activated by cytokines, NF-*κ*B enters the nucleus of cells where it binds to DNA to initiate gene transcription. There is potential for this binding to be inhibited by either blocking binding sites on the DNA itself or on NF-*κ*B. NF-*κ*B p65 DNA binding activity was assessed using a NF-*κ*B p65 transcription factor assay. Nuclear protein was then extracted. IL-1β induced a significant increase in NF-*κ*B DNA binding activity (Figure 4(b)). Co-treatment with both luteolin and kaempferol significantly attenuated IL-1β-induced NF-*κ*B p65 DNA binding activity (Figure 4(b)).

To examine the effect of luteolin and kaempferol on AP-1, we examined c-Jun, as it forms part of the AP-1 early response transcription factor [34]. When compared to untreated cells (basal), IL-1β induced an increase in c-Jun expression (Figure 4(c)). Coincubation with luteolin and kaempferol attenuated the IL-1β-induced increase in c-Jun nuclear protein expression (Figure 4(c)).

## 4. Discussion

The data presented in this study demonstrate that the two dietary phytophenols luteolin (from celery) and kaempferol (from grapefruit and tea) exert anti-inflammatory properties in gestational tissues. In human fetal membranes, luteolin and kaempferol treatment attenuated LPS, or IL-1β induced increases in mRNA expression and secretion of proinflammatory cytokines, COX-2 mRNA expression and subsequent prostaglandin release, and MMP-9 expression and secretion. In myometrium cells, luteolin and kaempferol significantly decreased COX-2 expression, prostaglandin release, and MMP-9 activity induced by IL-1β. There is, however, no effect of luteolin and kaempferol on proinflammatory cytokine expression or secretion in myometrial cells. Luteolin and kaempferol were found to act via the NF-*κ*B and AP-1 pathways, inhibiting NF-*κ*B p65 DNA binding activity and nuclear c-Jun expression in myometrial cells. 

Proinflammatory cytokines play a key role as mediators of inflammation in preterm and term labour. In human gestational tissues and amniotic fluid, these cytokines are increased with the onset of human labour at term [35], and preterm [36] and more so in the presence of infection [37]. TNF-*α* and IL-1β exert proinflammatory actions such as the increase of prostaglandins and ECM degrading enzymes [38]. This leads to initiation of the three critical stages of human labour: rupture of fetal membranes, cervical ripening, and uterine contractions [35, 39, 40]. In this study, we used human fetal membranes and myometrial cells to determine the effect of luteolin and kaempferol on LPS or IL-1β-induced expression and release of the proinflammatory cytokines TNF-*α*, IL-6, and IL-8. We found that both luteolin and kaempferol significantly reduced the mRNA expression and secretion of proinflammatory cytokines in fetal membranes. Similarly, past studies performed in nongestational tissues have reported such anti-inflammatory actions for luteolin and kaempferol [12, 15–17]. However, it was found that luteolin and kaempferol had no effect on cytokine expression or secretion from pregnant myometrial cells. It is possible that longer or shorter incubation times or higher concentrations of phytophenols are required to elicit a protective response in myometrial cells. 

COX-2 stimulates production of prostaglandins, which are important in the initiation and maintenance of labour, by increasing uterine contraction and promoting cervical ripening and the decidual-fetal membrane activation [38, 41]. Luteolin and kaempferol have been demonstrated in nongestational tissues to cause inhibition of COX-2, hence causing inhibition of prostaglandins [42, 43]. In this study, we demonstrate that luteolin and kaempferol significantly decreased LPS, IL-1β-induced COX-2 gene expression, and subsequent PGE_2_ and PGF_2*α*_ release in both fetal membranes and myometrial cells. This decrease in prostaglandin production could potentially detain the onset of uterine contraction and decrease the progression of cervical ripening, thus delaying the birth of the baby. 

MMP-9 plays a role in the myometrium during parturition. During pregnancy the uterus is remodeled and enlarged by the addition of collagen to the myometrium, to accommodate the growing fetus, placenta, and amniotic fluid [44]. MMP-9 degrades this collagen during parturition, shrinking the uterus [44]. In addition, MMP-9 plays a major role in the degradation of the collagen matrix within fetal membranes, causing weakening, which, along with stretch forces, leads to membrane rupture [45]. Consistent with previous studies in nongestational tissues [46, 47], both luteolin and kaempferol attenuated IL-1β-induced MMP-9 activity in primary cells from amnion and myometrium. Of note, in myometrium, IL-1β did not increase MMP-9 gene expression, nor was there attenuation with luteolin or kaempferol. This may be explained by the fact that there are two different types of MMP-9 in myometrium, pro-MMP-9 and active MMP-9. The MMP-9 which is important in the context of parturition is active MMP-9, as this is the active form of the enzyme. qRT-PCR cannot distinguish between the two forms of MMP-9 and so hence gives the total MMP-9 expression, not just the active MMP-9 expression. 

There is increasing evidence for the role of NF-*κ*B and AP-1 in human term and preterm labour [6–9, 48]. Furthermore, mouse studies have been employed to demonstrate that by inhibiting NF-*κ*B or AP-1, infection-induced PTB can be delayed [49, 50]. In nongestational tissues, luteolin and kaempferol are thought to exert their anti-inflammatory actions by inhibiting NF-*κ*B [12–15] and AP-1 [14, 16, 17]. Similarly, in this study we show that luteolin and kaempferol inhibited NF-*κ*B DNA binding activity. In addition, both luteolin and kaempferol inhibited IL-1β-induced c-Jun expression, which is a nuclear protein that is part of the AP-1 transcription pathway. These findings suggest that, in human gestational tissues, luteolin and kaempferol may exert their inhibitory effects on proinflammatory cytokines, COX-2, prostaglandins, and MMP-9 via NF-*κ*B. This is in agreement with previous studies, by our laboratory and others, that NF-*κ*B and AP-1 regulate the transcription of prolabour mediators in human gestational tissues [6–9, 25].

There is emerging evidence for phytophenols as therapeutic agents for a number of pathological conditions including cancer [51]. They are readily available, inexpensive and have multitargeted potential [51]. However, their potential as therapeutics has also been heavily debated. They show low bioavailability and lose function due to metabolic processing when given via dietary supplementation [52]. If given at nutritionally relevant concentrations, extensive deglycosylation, glucuronidation, sulfation, and methylation reactions occur, mediated by a range of enzymes in the small intestine, liver, and colon. It has been shown that pharmacological doses that saturate metabolic pathways are required to obtain the free form of these phytophenols in the blood [52]. Whether luteolin or kaempferol can be used as therapeutics to prevent or delay PTB must first be addressed using experimental animal models of infection or inflammation-induced PTB. However, of promise is a recent study which has shown that kaempferol increases gestational length in a pregnant mouse model [53].

In addition to the anti-inflammatory actions, phytophenols possess a wide range of biological activities. For example, they have shown that both luteolin and kaempferol also possess antioxidant, antimicrobial, and anticancer activities [42, 43]. They have also been shown to have cardioprotective, antidiabetic, and neuroprotective effects. Luteolin has also shown antiallergic activity *in vitro *and *in vivo *[43].

In conclusion, in this study, we demonstrate that luteolin and kaempferol inhibit prolabour and proinflammatory mediators in human gestational tissues. Both luteolin and kaempferol have demonstrated anti-inflammatory properties in gestational tissues, by inhibiting NF-*κ*B DNA binding activity, the AP-1 pathway, and their target genes. Given the central role of inflammation in provoking preterm labour, it is tempting to speculate that dietary phytophenols may be an effective, potential treatment or preventative for PTB. As a result of this research, further study is currently underway to determine effects of these phytophenols in an *in vivo* mouse model.

## References

[1] Howson CP, Kinney MV, Lawn JE (2012). *March of Dimes PfM, Newborn and Child Health, Save the Children, WHO. Born Too Soon: The Global Action Report on Preterm Birth*.

[2] Goldenberg RL, Culhane JF, Iams JD, Romero R (2008). Epidemiology and causes of preterm birth. *The Lancet*.

[3] Laws P, Sullivan EA (2009). *Australia's Mothers and Babies 2007*.

[4] Birth CoUP, Assuring Healthy Outcomes (2007). *Preterm Birth: Causes, Consequences, and Prevention*.

[5] Menon R, Dunlop AL, Kramer MR, Fortunato SJ, Hogue CJ (2011). An overview of racial disparities in preterm birth rates: caused by infection or inflammatory response?. *Acta Obstetricia et Gynecologica Scandinavica*.

[6] Lappas M, Rice GE (2007). The role and regulation of the nuclear factor *κ* B signalling pathway in human labour. *Placenta*.

[7] Lappas M, Rice GE (2009). Transcriptional regulation of the processes of human labour and delivery. *Placenta*.

[8] Mohan AR, Sooranna SR, Lindstrom TM, Johnson MR, Bennett PR (2007). The effect of mechanical stretch on cyclooxygenase type 2 expression and activator protein-1 and nuclear factor-*κ*B activity in human amnion cells. *Endocrinology*.

[9] Khanjani S, Terzidou V, Johnson MR, Bennett PR (2012). NF*κ*B and AP-1 drive human myometrial IL8 expression. *Mediators of Inflammation*.

[10] Havsteen BH (2002). The biochemistry and medical significance of the flavonoids. *Pharmacology and Therapeutics*.

[11] Middleton E, Kandaswami C, Theoharides TC (2000). The effects of plant flavonoids on mammalian cells: implications for inflammation, heart disease, and cancer. *Pharmacological Reviews*.

[12] Lv LH, Zhang YB, Kong QH (2011). Luteolin prevents LPS-induced TNF-*α* expression in cardiac myocytes through inhibiting NF-*κ*B signaling pathway. *Inflammation*.

[13] Kim S-H, Shin K-J, Kim D (2003). Luteolin inhibits the nuclear factor-*κ*B transcriptional activity in Rat-1 fibroblasts. *Biochemical Pharmacology*.

[14] Chen C-C, Chow M-P, Huang W-C, Lin Y-C, Chang Y-J (2004). Flavonoids inhibit tumor necrosis factor-*α*-induced up-regulation of intercellular adhesion molecule-1 (ICAM-1) in respiratory epithelial cells through activator protein-1 and nuclear factor-*κ*B: structure-activity relationships. *Molecular Pharmacology*.

[15] García-Mediavilla V, Crespo I, Collado PS (2007). The anti-inflammatory flavones quercetin and kaempferol cause inhibition of inducible nitric oxide synthase, cyclooxygenase-2 and reactive C-protein, and down-regulation of the nuclear factor *κ*B pathway in Chang Liver cells. *European Journal of Pharmacology*.

[16] Chen C-Y, Peng W-H, Tsai K-D, Hsu S-L (2007). Luteolin suppresses inflammation-associated gene expression by blocking NF-*κ*B and AP-1 activation pathway in mouse alveolar macrophages. *Life Sciences*.

[17] Choi EM, Lee YS (2010). Luteolin suppresses IL-1β-induced cytokines and MMPs production via p38 MAPK, JNK, NF-*κ*B and AP-1 activation in human synovial sarcoma cell line, SW982. *Food and Chemical Toxicology*.

[18] Lim DY, Cho HJ, Kim J, Nho CW, Lee KW, Park JHY (2012). Luteolin decreases IGF-II production and downregulates insulin-like growth factor-I receptor signaling in HT-29 human colon cancer cells. *BMC Gastroenterology*.

[19] Deqiu Z, Kang L, Jiali Y, Baolin L, Gaolin L (2011). Luteolin inhibits inflammatory response and improves insulin sensitivity in the endothelium. *Biochimie*.

[20] Markaverich BM, Vijjeswarapu M, Shoulars K, Rodriguez M (2010). Luteolin and gefitinib regulation of EGF signaling pathway and cell cycle pathway genes in PC-3 human prostate cancer cells. *Journal of Steroid Biochemistry and Molecular Biology*.

[21] Zhang Y, Liu D (2011). Flavonol kaempferol improves chronic hyperglycemia-impaired pancreatic beta-cell viability and insulin secretory function. *European Journal of Pharmacology*.

[22] Zhang Y, Chen AY, Li M, Chen C, Yao Q (2008). Ginkgo biloba extract kaempferol inhibits cell proliferation and induces apoptosis in pancreatic cancer cells. *Journal of Surgical Research*.

[23] Lin M-K, Yu Y-L, Chen K-C (2011). Kaempferol from Semen cuscutae attenuates the immune function of dendritic cells. *Immunobiology*.

[24] Bennett PR, Rose MP, Myatt L, Elder MG (1987). Preterm labor: stimulation of arachidonic acid metabolism in human amnion cells by bacterial products. *American Journal of Obstetrics and Gynecology*.

[25] Lappas M, Permezel M, Georgiou HM, Rice GE (2002). Nuclear factor *κ* B regulation of proinflammatory cytokines in human gestational tissues in vitro. *Biology of Reproduction*.

[26] Lappas M, Permezel M, Georgiou HM, Rice GE (2004). Regulation of Phospholipase Isozymes by Nuclear Factor-*κ*B in Human Gestational Tissues in Vitro. *Journal of Clinical Endocrinology and Metabolism*.

[27] Lappas M, Permezel M, Rice GE (2003). N-Acetyl-cysteine inhibits phospholipid metabolism, proinflammatory cytokine release, protease activity, and nuclear factor-*κ*B deoxyribonucleic acid-binding activity in human fetal membranes in Vitro. *Journal of Clinical Endocrinology and Metabolism*.

[28] Lappas M, Permezel M, Rice GE (2006). 15-Deoxy-Δ^12,14^-Prostaglandin J_2_ and troglitazone regulation of the release of phospholipid metabolites, inflammatory cytokines and proteases from human gestational tissues. *Placenta*.

[29] Livak KJ, Schmittgen TD (2001). Analysis of relative gene expression data using real-time quantitative PCR and the 2-ΔΔCT method. *Methods*.

[30] Lappas M, Odumetse TL, Riley C (2008). Pre-labour fetal membranes overlying the cervix display alterations in inflammation and NF-*κ*B signalling pathways. *Placenta*.

[31] Lindström TM, Bennett PR (2005). 15-Deoxy-*δ*12,14-prostaglandin J2 inhibits interleukin-1β-induced nuclear factor-*κ*B in human amnion and myometrial cells: mechanisms and implications. *Journal of Clinical Endocrinology and Metabolism*.

[32] Lanoix D, St-Pierre J, Lacasse A-A, Viau M, Lafond J, Vaillancourt C (2012). Stability of reference proteins in human placenta: general protein stains are the benchmark. *Placenta*.

[33] Scherer DC, Brockman JA, Chen Z, Maniatis T, Ballard DW (1995). Signal-induced degradation of I*κ*B*α* requires site-specific ubiquitination. *Proceedings of the National Academy of Sciences of the United States of America*.

[34] Udou T, Hachisuga T, Tsujioka H, Kawarabayashi T (2004). The role of c-jun protein in proliferation and apoptosis of the endometrium throughout the menstrual cycle. *Gynecologic and Obstetric Investigation*.

[35] Steinborn A, Geisse M, Kaufmann M (1998). Expression of cytokine receptors in the placenta in term and preterm labour. *Placenta*.

[36] Steinborn A, Günes H, Röddiger S, Halberstadt E (1996). Elevated placental cytokine release, a process associated with preterm labor in the absence of intrauterine infection. *Obstetrics and Gynecology*.

[37] Gonçalves LF, Chaiworapongsa T, Romero R (2002). Intrauterine infection and prematurity. *Mental Retardation and Developmental Disabilities Research Reviews*.

[38] Romero R, Mazor M, Munoz H, Gomez R, Galasso M, Sherer DM (1994). The preterm labor syndrome. *Annals of the New York Academy of Sciences*.

[39] Lee SY, Buhimschi IA, Dulay AT (2011). IL-6 trans-signaling system in intra-amniotic inflammation, preterm birth, and preterm premature rupture of the membranes. *Journal of Immunology*.

[40] Sennström MB, Ekman G, Westergren-Thorsson G (2000). Human cervical ripening, an inflammatory process mediated by cytokines. *Molecular Human Reproduction*.

[41] Bennett PR, Elder MG, Myatt L (1987). The effects of lipoxygenase metabolites of arachidonic acid on human myometrial contractility. *Prostaglandins*.

[42] Calderón-Montaño JM, Burgos-Morón E, Pérez-Guerrero C, López-Lázaro M (2011). A review on the dietary flavonoid kaempferol. *Mini-Reviews in Medicinal Chemistry*.

[43] López-Lázaro M (2009). Distribution and biological activities of the flavonoid luteolin. *Mini-Reviews in Medicinal Chemistry*.

[44] Roh C-R, Oh W-J, Yoon B-K, Lee J-H (2000). Up-regulation of matrix metalloproteinase-9 in human myometrium during labour: a cytokine-mediated process in uterine smooth muscle cells. *Molecular Human Reproduction*.

[45] Bou-Resli MN, Al-Zaid NS, Ibrahim MEA (1981). Full-term and prematurely ruptured fetal membranes. An ultrastructural study. *Cell and Tissue Research*.

[46] Yang H, Liu Q, Ahn JH (2012). Luteolin downregulates IL-1β-induced MMP-9 and-13 expressions in osteoblasts via inhibition of ERK signalling pathway. *Journal of Enzyme Inhibition and Medicinal Chemistry*.

[47] Shen SC, Lin CW, Lee HM, Chien LL, Chen YC (2006). Lipopolysaccharide plus 12-o-tetradecanoylphorbol 13-acetate induction of migration and invasion of glioma cells in vitro and in vivo: differential inhibitory effects of flavonoids. *Neuroscience*.

[48] Allport VC, Slater DM, Newton R, Bennett PR (2000). NF-*κ*B and AP-1 are required for cyclo-oxygenase 2 gene expression in amnion epithelial cell line (WISH). *Molecular Human Reproduction*.

[49] Pirianov G, Waddington SN, Lindström TM, Terzidou V, Mehmet H, Bennett PR (2009). The cyclopentenone 15-deoxy-*δ*12,14-prostaglandin J 2 delays lipopolysaccharide-induced preterm delivery and reduces mortality in the newborn mouse. *Endocrinology*.

[50] Nath CA, Ananth CV, Smulian JC, Peltier MR (2010). Can sulfasalazine prevent infection-mediated pre-term birth in a murine model?. *American Journal of Reproductive Immunology*.

[51] Gupta SC, Kim JH, Prasad S, Aggarwal BB (2010). Regulation of survival, proliferation, invasion, angiogenesis, and metastasis of tumor cells through modulation of inflammatory pathways by nutraceuticals. *Cancer and Metastasis Reviews*.

[52] Duthie GG, Gardner PT, Kyle JAM (2003). Plant polyphenols: are they the new magic bullet?. *Proceedings of the Nutrition Society*.

[53] Johnson JR, Makaji E, Ho S, Xiong B, Crankshaw DJ, Holloway AC (2009). Effect of maternal raspberry leaf consumption in rats on pregnancy outcome and the fertility of the female offspring. *Reproductive Sciences*.

